# Photocell-Based Optofluidic Device for Clogging-Free Cell Transit Time Measurements

**DOI:** 10.3390/bios14040154

**Published:** 2024-03-24

**Authors:** Filippo Storti, Silvio Bonfadini, Gaia Bondelli, Vito Vurro, Guglielmo Lanzani, Luigino Criante

**Affiliations:** 1 Centre for Nano Science and Technology, Istituto Italiano di Tecnologia, Via Rubattino 81, 20134 Milano, Italy; filippo.storti15@gmail.com (F.S.); silvio.bonfadini@iit.it (S.B.); gaia.bondelli@gmail.com (G.B.); vito.vurro2@unibo.it (V.V.); guglielmo.lanzani@iit.it (G.L.); 2 Dipartimento di Fisica, Politecnico di Milano, Piazza Leonardo da Vinci 32, 20133 Milano, Italy

**Keywords:** cell transit time, transit time measurement, optofluidic, FLICE, clogging-free, cell deformability, MCF-7, cancer cells

## Abstract

Measuring the transit time of a cell forced through a bottleneck is one of the most widely used techniques for the study of cell deformability in flow. It in turn provides an accessible and rapid way of obtaining crucial information regarding cell physiology. Many techniques are currently being investigated to reliably retrieve this time, but their translation to diagnostic-oriented devices is often hampered by their complexity, lack of robustness, and the bulky external equipment required. Herein, we demonstrate the benefits of coupling microfluidics with an optical method, like photocells, to measure the transit time. We exploit the femtosecond laser irradiation followed by chemical etching (FLICE) fabrication technique to build a monolithic 3D device capable of detecting cells flowing through a 3D non-deformable constriction which is fully buried in a fused silica substrate. We validated our chip by measuring the transit times of pristine breast cancer cells (MCF-7) and MCF-7 cells treated with Latrunculin A, a drug typically used to increase their deformability. A difference in transit times can be assessed without the need for complex external instrumentation and/or demanding computational efforts. The high throughput (4000–10,000 cells/min), ease of use, and clogging-free operation of our device bring this approach much closer to real scenarios.

## 1. Introduction

The mechanical properties of single cells can provide useful information about their physiological state or the pathological processes they may have undergone. For this reason, the assessment of cell deformability promises to be a captivating, label-free biomarker for the identification of different cell types or diseases [1]. The applications include the measurement of stiffness changes caused by illnesses such as diabetes [2], sepsis [3], and malaria [4,5]. Applied studies in the field of cancer diagnosis have also highlighted that the variations in deformability significantly distinguish the malignancy and invasiveness of tumoral cells [6]. Moreover, the drug responses of cancer cells can be monitored by assessing the mechanical properties of individual cells, potentially supporting a personalized medicine approach [1,7]. Many techniques have been implemented to investigate single-cell mechanical features, including atomic force microscopy [8], optical stretching [9], optical [10] or magnetic [11] tweezers, and micropipette aspiration [12,13,14]. As many of these tools analyze each cell individually, the throughput is intrinsically limited to <1 cell/min [15], which hinders their use in clinical or research settings. In addition, complex instrumentation and highly trained operators are required in order to perform accurate measurements. Therefore, the translation to diagnostic-oriented devices is still an open challenge. Alternatively, microfluidic platforms allow for easier handling of single cells. The ideal device should implement a “deformability cytometry” (DC) [1] featuring high throughput—approaching normal flow cytometers (~1000 cells/s)—and automation, thus minimizing user involvement. Currently, microfluidics-based methods vary in the type of deformation applied and the evaluation strategy. Three main classes can be identified, according to the stress applied to deform a single cell: shear stress DC (sDC), extensional flow DC (xDC), and constriction-based DC (cDC) [16]. In the first two cases, the cell shape is elongated in a contactless manner by exploiting the high shear stresses developed using a long, narrow channel, which is wider than the cell diameter [17], or using the hydrodynamic forces induced by the crossing of two flows in a cross-shaped microfluidic network [18,19]. The accuracy of estimating the Young’s modulus of the cells is high thanks to studies that have linked experiments and numerical simulations to analytical theory [20,21], as is the throughput of these devices, ranging from 100 cells/s to 2000 cells/s [22]. However, the need for expensive and bulky external instrumentation to achieve accurate analysis remains the main drawback. Indeed, a high-speed camera coupled to a microscope objective is required in order to characterize the deformation. In addition, the analysis relies entirely on image processing, which tends to be time-consuming and computationally expensive, precluding the possibility of real-time analysis.

Instead, the cDC method exploits the flow of individual cells through a constriction that is narrower than the cell’s diameter, thereby deforming it. In general, cell deformability is mainly related to the time required to travel through the constriction, referred to as the transit time [16]. This strategy allows for robust assessment of mechanical properties and combines the effect of cells’ stiffness with that of their size. In fact, smaller cells have shorter transit times, as they are less affected by the channel bottleneck. This can be of great advantage in identifying different classes of cells, and could help to implement a screening technique to detect spiked tumoral cells in biological samples [23,24]. Besides the high-speed camera method [25,26,27,28], other techniques to measure the transit time include the change in the impedance across two integrated electrodes [24,29,30,31,32,33] and the variation in the resonant frequency in a suspended microfluidic resonator (SMR) caused by the cell passage in the channel [23]. Unfortunately, impedance-based methods can be susceptible to environmental changes, such as temperature, or to variations in the chemical composition, and, hence, the electrical conductivity, of the flowing medium, while the SMR approach is still relies on the external setup to align an optical beam in order to detect the cantilever displacement. Hence, in this work, we propose an optofluidic method by integrating two pairs of optical fibers in a microfluidic channel to measure the transit time across the channel restriction. The proposed solution offers important advantages over the state of the art. Firstly, the use of photonics in analytical systems generally exhibits superior performance in terms of both immunity to external interferences (robustness) and time response. Thanks to the proposed manufacturing technique—femtosecond laser irradiation followed by chemical etching (FLICE)—the constriction can be three-dimensional and buried inside a non-deformable substrate (fused silica), unlike the majority of works in the literature based on lithography, which use polymeric (soft) materials, e.g., polydimethylsiloxane (PDMS), and 2D bottlenecks [24,25,26,27,28,29,30,31,32,33]. Finally, the length of the constriction can be very short (a few tens of microns), allowing the study to focus exclusively on cell deformation and to neglect the friction effect. All of this guarantees the achievement of high-pressure—and, therefore, high-throughput—clogging-free working conditions without deforming the bottleneck or requiring any bulky external vision system. The proposed strategy has been characterized by tests with both polystyrene microbeads (PS) and breast cancer cells (MCF-7). Finally, the stiffness of MCF-7 cells was modified using the drug Latrunculin A to detect variations in the transit times of untreated versus treated cells.

## 2. Materials and Methods

### 2.1. Device Design

**Figure 1:**
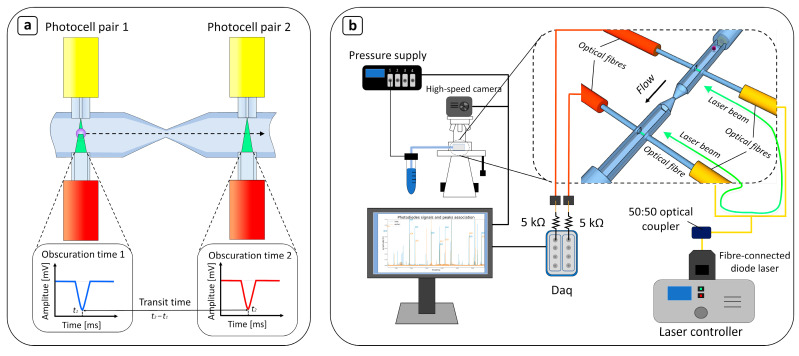
Schematic diagram of the device. (**a**) The device’s working principle is based on inducing deformations in flowing cells by forcing them to pass through a constriction (12 μm × 20 μm × 40 μm, width × height × length) of the main channel (100 μm × 100 μm × 3200 μm). The deformability is inversely proportional to the time taken by the particle to pass through the narrowing, i.e., the transit time [16]. To measure this, two pairs of optical fibers are inserted opposite each other and orthogonal to the flow direction. This creates two photocell-like regions before and after the bottleneck, with their axes of symmetry perfectly aligned with the center of the channel. Each pair of fibers measures the variation in a constantly shined laser beam, induced by the passage of a cell. Each passing object is associated with a downward peak in the received optical signal, the width of which is called the “obscuration time”. By computing the shift between the first and the second minimum of the obscuration time peak, it is possible to extract the transit time of the flowing object. (**b**) Microfluidic and data acquisition setup: Tests were also carried out on the optical microscope with the aid of a high-speed CCD at 8000 fps to endorse the correct operation of the device’s transit time measurement, but its operating principle is designed to be independent of the vision setups.

Our device implements a cDC to measure the transit time required for a cell to flow through a 3D microfluidic constriction. When considering the passage of a cell through a narrow channel—typically ranging in length from 80 μm to 500 μm and in width from 6 μm to 10 μm—two main effects need to be considered: cell deformability/cell size and the “contactless friction” between the cell envelope and the microchannel walls. In the initial phase, called the “entry phase”, the cell (diameter 15–30 μm) significantly reduces its dimensions to enter the constriction, so its elasticity plays a key role. Instead, the cell does not change shape as it flows through the constriction, and the friction developed between the cell surface and the channel walls dominates [23,24,34]. To focus on the cell deformation and neglect the friction effect, our device presents a 3D constriction (12 μm wide, 20 μm high) that is 40 μm long, which is shorter than the devices in most works found in the literature (>100 μm). Moreover, by reducing the constriction length, the risk of clogging the bottleneck is also reduced. Two pairs of optical fibers positioned orthogonally to the mainstream (Figure 1a) allow for the measurement of the transit time across the channel shrinkage. The geometry of the microfluidic circuit is very compact, with a total length of 3.2 mm. In addition, thanks to the peculiarities of our manufacturing technique, the channel shrinks not only in the xy plane, but also in the zx plane (Figure 1b). The main channel narrows along the *z*-axis, reducing its height from 100 μm to 50 μm to prevent more than one cell from passing through the photocell area at the same time, and the optical fibers are self-aligned by their micro-machined housings. To smooth the transition from the main channel (sized 100 μm × 50 μm) to the constriction, the two parts are connected by a conical constriction area 200 μm in length. The geometry is completed by four cylindrical housings that allow the optical fibers to be placed directly into contact with the fluid flowing in the main channel. The two optical fibers of the single pair face each other, constituting a photocell-like element that detects objects flowing in the channel. When a cell crosses the light beam in the detection segment, the optical signal is both scattered and diffracted so that part of it is no longer included in the acceptance cone of the acquisition fiber. This creates a signal drop in the photocell output, and the time difference between the peaks of the first and the second fiber pair gives the transit time of the cell through the constriction (Figure 1a).

### 2.2. Device Fabrication

The microfluidic circuit is entirely fabricated using the FLICE technique [35,36,37,38,39], which is based on two main processes. In the first step, an ultrashort-pulsed laser beam irradiates a fused silica substrate according to the 3D designed geometry. Due to non-linear absorption effects, the chemical–physical properties of the material are modified only in the region confined to the beam spot volume. The second step, wet etching, consists of a bath in a chemical solution that allows for the selective removal of the modified material. The result is a robust, monolithic network of hollow microchannels that is completely buried in the substrate. The chemical solution used in this work is a water-based solution of hydrofluoric acid (HF) at a concentration of 20%. Thus, the fabrication of a compact device in a non-deformable substrate is possible, with considerable benefits regarding the aim of the present study. In fact, the use of fused silica as the main constituent material offers a twofold advantage. Firstly, its Young’s modulus (tens of GPa) is 3 orders of magnitude greater than that of the cells, preventing any possible deformation of the bottleneck as the cells pass through. This, in turn, allows for the use of a high inlet pressure, which achieves a high throughput and reduces the risk of clogging, even with clusters of cells. Since the bottleneck must meet strict size constraints, the fabrication is optimized to reduce the etching times and increase the spatial resolution. To reach higher etching rates, the writing parameters are set to align the nanostructures induced by the femtosecond laser irradiation along the etching direction [40]. The accuracy of the constriction dimensions is then obtained by fabricating it using a single line of modified material (with a resolution of 3–5 μm), attenuating the delivered optical power of 30%. The rest of the designed geometry is filled with lines of unmodified material, spaced 7 μm apart to optimize the writing time. The laser beam irradiates the substrate with an average power of 200 mW (400 nJ), scanning the desired pattern at a speed of 1 mm/s. The writing process takes 1.5 h to complete, while the chemical etching takes approximately 2 h.

The device is then completed by inserting and fixing it into the relatives’ housing with UV-glue, two single-mode optical fibers (P1-460Y-FC, Thorlabs, Newton, NJ , USA, 4 μm core and 0.14 NA), two multimode fibers (M14L, Thorlabs, with 50 μm core and 0.22 NA), and PEEK microtubes (UP-1572, Microcolumn, Desio (MB), Italy).

### 2.3. Device Characterization

To optimize the working pressures and validate the transit time extraction algorithm, preliminary experiments were carried out using a water-based solution containing PS microbeads of different sizes—from 10 to 20 μm in diameter—at different inlet pressures ranging from 40 to 200 mbar. The inlet pressures were precisely controlled by means of a pressure injection system (OB1MK3) supplied by Elveflow, Paris, France. As PS particles are non-deformable, a suitable bottleneck was designed for this preliminary test. The device used for these experiments had a 30 μm widened constriction, which allowed the effect of the particle size to be analyzed.

After completing the test/calibration, we investigated the capabilities of the device to operate with deformable particles by analyzing living cells. Unlike beads, cells are semi-transparent objects and give rise to peculiar obscuration signals when passed through a light beam. Therefore, the transit time detection algorithm was adapted to living cells, using MCF-7 cells, a type of breast cancer, as a biological model. The ability of the device to detect a change in cell deformability by measuring the transit time was evaluated by processing two sub-populations of MCF-7 cells. One sub-population consisted of pristine cells, while the other comprised cells that had been treated with a specific drug (Latrunculin A) to disrupt actin filaments in the cytoskeleton, making them more compliant.

The excitation light was provided by a single-mode, pigtailed, in-fiber laser diode (LP520-SF15, Thorlabs) connected to the single-mode optical fibers by a 50:50 coupler (TW560R5F1, Thorlabs). The shined laser beam (λ = 520 nm) hit the facets of multimode fibers constantly during the experiment. The harvested optical signal was converted into electrical signals by two fast Si photodiodes (2 GHz bandwidth) (DET025AFC/M, Thorlabs) sampled at 100 kHz using a National Instruments (Austin, TX, USA) 16-bit data acquisition (DAQ) card—up to 3.5 MS/s/ch—and analyzed using a customized LabVIEW interface (National Instruments, LabVIEW 2017). To convert the photocurrent generated by the photodiodes into an output voltage, two load resistors were added to the connection between the photodiodes and the DAQ card. The resistances value was set to 5 kΩ to provide the best trade-off between the system bandwidth and the absolute channel voltage response. In this way, the multimode acquisition fiber could see a constant optical signal when there was no particle fluid in the photocell area, but when a cell passed through, it scattered and diffracted the light from the fiber, causing the signal to decrease as long as the cell intercepted the beam. Since the two pairs of photocells began recording at the same time, both providing an electrical signal, the transit time was obtained by computationally extracting the shift between the obscuration peaks in the two signals. The resolution concerning the obscuration time measurement was about 1 μs (see Supplementary Materials for more details (Figure S1)). Microfluidics experiments were performed under an optical microscope (BX53M, Olympus, Tokyo, Japan) connected to a high-speed camera (FASTCAM Mini UX100 type 800k-M-16G, Photron, Tokyo, Japan) in order to be able to characterize the fluidic behavior of the device as well, and to validate the results of the device measurements with high-speed videos (8000 fps, 1 s duration). To ensure good visualization of the flowing cells, the working inlet pressure was set to 50 mbar for all the experiments involving MCF-7 cells. However, it is important to highlight that the device was designed to work independently of any vision system.

### 2.4. Cell Preparation

Measurements were performed using breast adenocarcinoma cells (MCF-7) purchased from ATCC. Cells were cultured in T-25 cell culture flasks containing Dulbecco’s modified eagle medium—high glucose (DMEM-HG) supplemented with 10% heat-inactivated fetal bovine serum (FBS, Gibco, Paisley, UK), 2% penicillin/streptomycin, and 1% L-glutamine. Culture flasks were maintained in a humidified incubator at 37 °C with 5% CO_2_ to preserve physiological conditions. When the cells reached 80% confluence, they were enzymatically detached with a 1× trypsin-EDTA solution, centrifuged (5 min, 1200 rpm), and resuspended in fresh DMEM previously filtered through a 0.22 µm PVDF filter to prevent clogging of the microfluidic system. MCF-7 cells were made more compliant through a 60 min treatment with Latrunculin A 0.1 µM (Invitrogen, Thermo Fisher Scientific, Waltham, Massachusetts, USA). This is a toxin known to disrupt actin filaments, thereby reducing the elastic modulus of the cells. The sizes of the suspended cells were checked via microscopy and found to be in the range of 15–30 µm. Prior to injection into the microfluidic circuit, the cells were filtered through a Falcon cell strainer with 40 μm pores (Corning, New York, NY, USA; catalogue number: 352340) to help to reduce the number of cell clusters.

### 2.5. Data Analysis

The acquired data plotted the intensity of the optical signal seen by the multimode fibers versus time. Each time a cell (or a cluster of them) crossed the optical beam, a decrease in the signal intensity was recorded, resulting in downward peaks. For this reason, a custom peak detection algorithm was implemented using MATLAB (MATLAB, 2018, version R2018b, The MathWorks Inc., Natick, MA, USA). To associate the signal with the “cell passage” event, the peak was required to fulfill several requirements in terms of both its shape (a maximum of two lobes are allowed) and its duration. This was necessary in order to select only the events associated with the passage of a single cell and to discard the others, i.e., the passage of clusters or air bubbles. Indeed, clusters of cells can be composed of an unknown number of cells, which can vary greatly between different clusters.

Therefore, the analysis of individual cells provides a more robust description of the deformability status of the processed population. After peak detection, transit time extraction was performed by selecting, for each photocell peak before the bottleneck, the first matching photocell peak after the bottleneck, which was required to occur within a reasonable time interval after the first photocell peak. A more detailed description of the transit time extraction step can be found in the Supplementary Materials (Figure S2).

## 3. Results and Discussion

### 3.1. Polystyrene (PS) Particles

**Figure 2:**
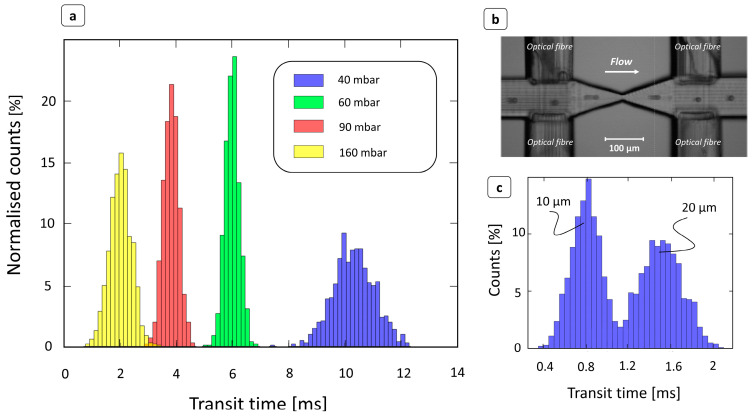
Characterization of devices using PS beads. (**a**) Transit time distributions at different inlet pressures for PS beads 15 μm in diameter dispersed in a deionized water solution. As the injection pressure increased, the particle velocity also increased, and the time to flow through the constriction area decreased. (**b**) Image obtained by superimposing different frames showing a PS bead flowing through the channel. The video was captured at 8000 fps through a 10× objective. (**c**) Transit time distribution in case of a water solution of PS beads of mixed size (10 μm and 20 μm) at 200 mbar. Particles with smaller diameters flowed at a higher velocity [41] and therefore had shorter transit times. The device was able to correctly detect two superimposed time distributions corresponding to the size difference.

The device operating principle was preliminarily evaluated by processing a water-based solution of PS beads. Obviously, PS particles are not deformable, but these tests were useful to properly characterize the optical signal detection configuration and to validate the transit time extraction algorithm. Figure 2a shows, for each different inlet pressure ranging from 40 to 160 mbar, the transit time distribution extracted by the device in the case of flowing beads 15 μm in diameter. Here, the transit times showed a typical Gaussian distribution, in the width was affected by the inlet pressure. Although the particles were approximately the same size, the higher variance displayed, particularly at low pressures, was mainly due to the different trajectories taken by the particles as they passed through the photocell beam.

As the flow field velocity increased, due to an increase in inlet pressure, the transit times accordingly shifted to lower values. This result ensures the correct functioning of the device, as well as the correct operation of the peak detection algorithm. The relationship between size and transit time was also investigated by flowing a water-based solution of 10 and 20 μm particles. As expected, smaller beads showed shorter transit times, because their velocities were higher than those of larger particles [41]. Indeed, in Figure 2c, the transit time distribution is clearly divided into two main populations, which can be related to the two different particle sizes.

### 3.2. MCF-7 Cells

**Figure 3:**
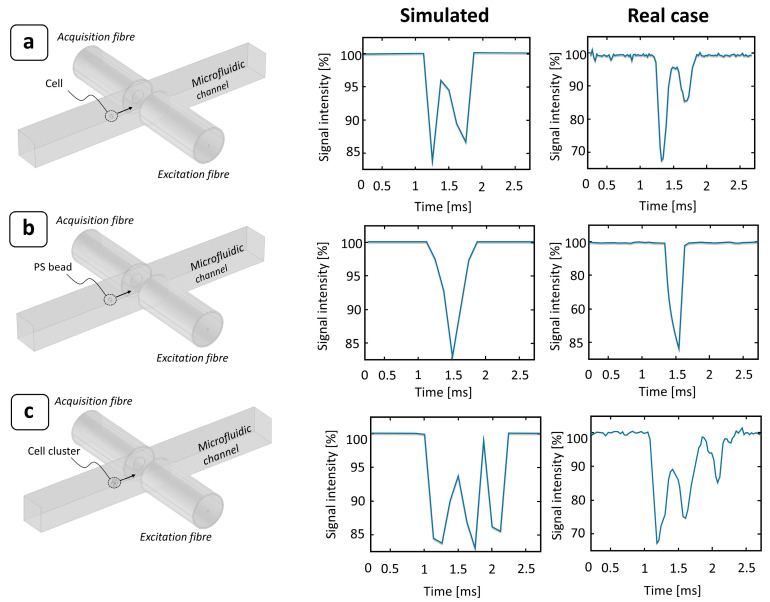
Effect of a particle passing through a photocell region: comparison between numerical ray-tracing simulations and experimental signal obtained under different conditions. The flowing particle was assumed to be spherical and to pass at the center of the microfluidic channel. The surrounding medium was modeled as DMEM (n_m_ = 1.33) [44], while the particle was considered as (**a**) a single MCF-7 cell (n_c_ = 1.4) [42]; (**b**) a single PS bead (n_PS_ = 1.6) [43]; and (**c**) an MCF-7 cell cluster (6 cells).

To obtain a complete picture of the device operation, a transit time analysis of deformable objects was performed, using MCF-7 cells as a biological model for deformability assessment. To accurately determine the passage of a cell through the photocell region, a tailored detection algorithm was implemented (see Supplementary Materials—paragraph 1). In contrast to PS microbeads, which are optically homogeneous objects, single cells are complex systems composed of several parts, which are often optically anisotropic. This produces a characteristic double-peak signal due to their greater optical transparency (Figure 3a). In the case of PS beads, the optical signal was almost completely scattered and diffracted by the particle while passing through the beam. Conversely, since the refractive index difference between the cells (n_c_ = 1.4) [42] (or PS beads [43]) and the medium (n_m_ = 1.33) [44] was small, the optical signal was significantly scattered or diffracted when crossing the interfaces of the different cell layers, i.e., the round meniscus of the membrane, whereas it was almost completely transmitted when hitting the cell body. This effect was well represented by numerical simulations and confirmed by experimental observation, as shown in Figure 3.

**Figure 4:**
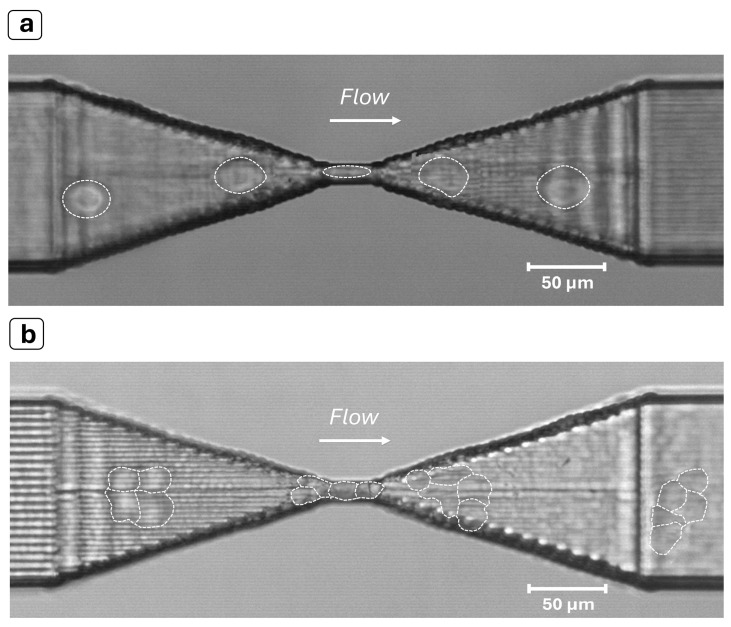
Experiments with MCF-7 cells. (**a**) Image of a cell (see dashed shape) squeezing through the constriction. (**b**) Cluster of MCF-7 cells (see multiple dashed shape) flowing through the constriction despite its size being significantly larger than the narrowing. The device shows a remarkable self-unclogging behavior thanks to the much higher Young’s modulus of the bulk substrate (fused silica) with respect to the flowing objects and the constriction length, which is significantly shorter than is usually found in the literature. The images were obtained by superimposing successive frames of a video recorded at 8000 fps with a 20× objective.

MCF-7 cells are a type of breast cancer cell, and their nature is to create inter-membrane adhesions with other cells to develop growing agglomerates. Therefore, when suspended in a medium, random collisions between cells may generate clusters composed by multiple particles, typically in the range of 2 to 10. This is often a limiting factor in constriction-based deformability assessment, since it can lead to the complete jamming of the channel. As the length of the constriction increases, so does the risk of a cluster of a few cells becoming stuck as it flows through. For this reason, our device was designed with a short bottleneck (about 40 μm), enough to contain 1 or 1.5 times the cell size and, thus, to allow for significant cell deformation. In this way, both single cells and clusters of them can pass through the constriction without affecting the device’s operability (Figure 4). Like the single cells, their clusters also provide characteristic optical signals, with longer obscuration times and multi-peak shapes (Figure 3c). This characteristic makes it possible to detect the presence of agglomerates and discard them from the final transit time extraction to obtain a clean single-cell analysis.

**Figure 5:**
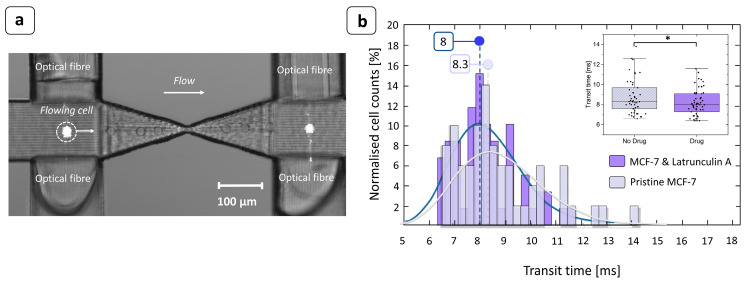
MCF-7 transit time measurement. (**a**) Overlapping of successive frames taken at 8000 fps showing the photocell-like behavior of the device. The cell flowing in the channel clearly crossed the light beam emitted by the optical fiber and interrupted the signal acquired by the opposite optical fiber, allowing the transit time to be accurately measured. The image was taken using an optical microscope with 10× objective. (**b**) At an injection pressure of 50 mbar, the shift between the two medians (continuous curves) of the transit time distributions was 0.3 ms. Since Latrunculin A acted on the cell deformability, increasing it, the transit time required to pass through the constriction was shorter than for untreated cells. Inset: the box plot of the transit times with and without the presence of the drug. The median transit time of the MCF-7 cells exposed to Latrunculin A was significantly lower than that of unexposed cells ( * Mann–Whitney U test, *p*-value < 0.05).

The capability of the device to detect changes in cell deformability strictly related to the transit time measurements was tested by processing two sub-populations of MCF-7 cells (Figure 5): one treated with the Latrunculin-A drug to increase cell deformability and the other untreated. The comparison of the transit time distributions between the two cases at the same inlet pressure clearly shows a difference between the two sub-populations. MCF-7 cells treated with Latrunculin-A had significantly shorter transit times (Mann–Whitney U test, MATLAB, *p*-value < 0.05), which is linked to their increased deformability. At an inlet pressure of 50 mbar, the shift between the two populations of transit times was 0.3 ms (Figure 5b). The histogram was constructed by considering 10 tracks 1 min each in length for the drug-treated case and 10 tracks for the pristine case. Out of these tracks, only clean single peaks that could be associated with a single cell passage were examined. The total numbers of cells considered were 59 cells for MCF-7 treated with Latrunculin A and 54 for untreated cells. This result is in good agreement with the high-speed videos recorded during the experiments, which showed a shift between the two populations of transit times in the range of 0.25 ÷ 0.30 ms, as is consistent with the results reported in the literature [33]. The experiments were carried out under highly diluted conditions (20,000 cells/mL) in order to reduce the frequency of events associated with cell clusters. At this injection pressure, the measured transit times ranged from 6 to 14 ms, which could potentially lead to high analysis throughputs of 4000–10,000 cells/min. This fully integrated optical detection system frees this type of analysis from the use of bulky or expensive external instrumentations and the alignment required to use them. It is also noteworthy that the data processing algorithm is remarkably lean, requiring less processing time than methods based on high-frame-rate vision systems. All these features give the proposed analysis strategy robustness, automation, and portability, making it a suitable solution for implementing a deformability-based cytometer that is applicable to clinical routine.

## 4. Conclusions

The assessment of cells’ mechanical properties is very promising in clinical applications due to the large amount of information that can be extracted. In the field of “deformability cytometry” (DC), constriction-based DC is one of the most promising methods for the further development of diagnostics-oriented devices. Its operating principle is based on measuring the time it takes the cell to pass through a bottleneck. An innovative, vision-less solution that couples optical analysis with microfluidics and integrates the whole optofluidic circuit on a single fused silica platform of a few millimeters (~3 mm) to perform transit time analysis is shown here. The device implements photocell-like time measurements of the cell through a constriction shorter than the usual lengths found in the literature in order to measure cell compliance alone. The optical detection of cell passage is completely independent of the medium conditions and electromagnetic noise and allows for high throughputs of potentially more than 10,000 cells/min. The latter is crucially determined using the FLICE technique, which exploits the mechanical properties of a robust and inert substrate—fused silica—by providing non-deformable 3D constriction channels. Especially when dealing with tumoral cells, clusters of cells can flow in the microfluidic circuit, which usually leads to jamming issues. Instead, thanks to its shorter bottleneck combined with its high structural Young’s modulus, the presented device instead shows impressive self-unclogging capabilities. As a proof of concept, MCF-7 cells were used to measure the transit times under two different conditions, involving pristine cells in the first and cells treated with Latrunculin A to increase their deformability in the second. As expected, the distribution of transit times shifted towards shorter values in the latter case, with a median value 0.3 ms faster than in the pristine case. Although, theoretically, the transit time obtained as the time difference between two events may be infinitesimal, experimentally, the resolution of our chip has been proven to be between 0.2 and 0.3 ms (see Supplementary Materials for more detail—paragraph 2). Particularly at low inlet pressures, the variance shown in the transit time (characterized by the width of the interpolation Gaussian) can sometimes still be too high, significantly reducing the device’s ability to analyze small differences in the elasticity of different cell types, mainly due to the different trajectories assumed by the particles as they pass through the beam. A microfluidic alignment section, such as hydrodynamic focusing [45], integrated immediately before the constriction can contribute to a decisive optimization of such cell deformability studies. However, thanks to the simplicity of operation and the robustness of our device, the processing of the data to extrapolate the transit time distribution requires minimal computational effort. Taken together, these features bring the proposed strategy closer to routine clinical-oriented applications.

## Data Availability

The data that support the findings of this study are available from the corresponding author upon reasonable request.

## Supplementary Materials

The following supporting information can be downloaded at: https://www.mdpi.com/article/10.3390/bios14040154/s1. Figure S1: To ensure that the peaks seen by the multimode acquisition fibres are single flowing cells, a double threshold is applied to the obscuration times. Experimentally, we have found that obscuration times below 0.5 ms are too fast to be cells and may be related to small cell debris present in the solution, while obscuration times above 1.3 ms are related to clusters of cells or flowing objects larger than a single cell size; Figure S2: The transit time computed between two consecutive peaks in the output signal of the two photocells. To correctly identify two subsequent events and associate them with the passage of a cell through the bottleneck, an acceptance window must be defined. This eliminates both overlapping peaks–i.e., when only the second photocell detects a flowing cell–and peaks that are not correlated–i.e., when only the first photocell detects cell passage.
