# Supplementary material for: Photocell-Based Optofluidic Device for Clogging-Free Cell Transit Time Measurements
Source: Biosensors (Basel). 2024 Mar 24;14(4):154. doi: 10.3390/bios14040154 (PMC11047832; doi:10.3390/bios14040154)
Supplement: Supplementary file 1 [file biosensors-14-00154-s001.zip › biosensors-2866780-supplementary.pdf]

biosensors

MDPI

Supporting Information

Photocell-based optofluidic device for clogging-free cell transit time measurements

Filippo Storti [1,2], Silvio Bonfadini [1], Gaia Bondelli [1,2], Vito Vurro [1,3], Guglielmo Lanzani [1,2] and Luigino Criante [1,*]

1 Centre for Nano Science and Technology, Istituto Italiano di Tecnologia, Via Rubattino 81, 20134 Milano, Italy
2 Politecnico di Milano, Dipartimento di Fisica, Piazza Leonardo da Vinci 32, 20133 Milano, Italy
3 Dipartimento di Fisica e Astronomia, Università di Bologna, Viale Berti Pichat 6”2, 40127 Bologna, Italy
* Correspondence: luigino.criante@iit.it;

Citation: To be added by editorial staff during production.

Received: 25 January 2024
Revised: 12 March 2024
Accepted: 21 March 2024
Published: 24 March 2024

Copyright: © 2024 by the authors. Submitted for possible open access publication under the terms and conditions of the Creative Commons Attribution (CC BY) license (https://creativecommons.org/licenses/by/4.0/).

1. Transit time extraction algorithm

To correctly identify and extract cell transit times, the raw data is screened to remove unwanted events such as large cell clusters or air bubbles. The procedure is as follows:

- **Pre-processing to facilitate peak extraction:** starting from the temporal behavior of the electrical signal acquired downstream of the photodetectors, the baseline (the signal acquired in the absence of the participle passage) is extrapolated and subtracted from the graph; the data are then inverted and the negative values are set to zero.

- **Filtering:** As shown in Figure 3, due to the special diffraction behavior of a transparent object with a spherical-like geometry, the obscuration of an optical beam typically results in a double-peaked peak. To aid the subsequent transit time extraction, the signal, is scanned by a root-mean-square envelope filter (sliding window = 5 samples) to smooth multi-peaks into single peaks instead. This procedure facilitates automatic peak detection to associate each peak with a “cell passage” event.

- **Peak detection:** peaks are found by setting an amplitude threshold, i.e. the peak intensity must be greater than five times the standard deviation of the signal. To avoid detecting multiple peaks that may be left over from the previous step, the number of samples between two single peaks must be twice the maximum average obscuration time – i.e. the time during which the photocell beam is scattered and refracted by the crossing cell. This time is determined experimentally, also according to the baseline width of each peak related to the "cell passage" event, and the value is set at 1.3 ms. The width of each peak is then computed to detect non-single cell passages, as described in the next step.

- **Deleting unwanted events:** Tumoral cells have a natural tendency to aggregate and form clusters of cells. Pre-measurement filtering greatly reduces this problem but does not eliminate it. It is therefore necessary to remove all the signals associated with these undesirable events to avoid misinterpretation of the analysis. If the object crossing the laser beam of the photocell is made up of several cells sticked together, the signal obtained will generally have a longer obscuration time. In addition, little debris can be usually found in biological samples which may give very short obscuration times. For these reasons, both a maximum and a minimum signal width threshold are set, to select only the peak suitable for the transit time calculation. The minimum width threshold is set at 0.5 ms while the maximum is again set at 1.3 ms, both determined experimentally (Fig. S1).

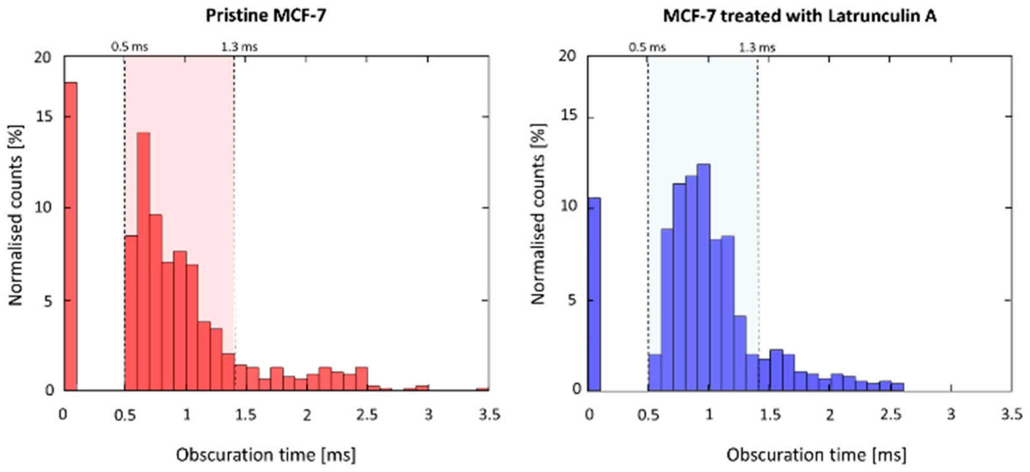

Figure S1. To ensure that the peaks seen by the multimode acquisition fibres are single flowing cells, a double threshold is applied to the obscuration times. Experimentally, we have found that obscuration times below 0.5 ms are too fast to be cells and may be related to small cell debris present in the solution, while obscuration times above 1.3 ms are related to clusters of cells or flowing objects larger than a single cell size.

- **Transit time extraction:** as both photocells signal recordings start simultaneously, the peak position in time of the first photocell signal is reported on the second track. From this point, a selection window (width = 15 ms) is defined to identify the corresponding peak on the second photocell recording. The width chosen corresponds to the inlet pressure set for the experiments. This value is inversely proportional to the flowing object speed. The window is placed at 1/10 of its width from the peak abscissa (fig. S2), in order to avoid an overlap between the peaks of the two tracks, which would be physically impossible according to the operating logic of the device. If a peak is found within the selection window, the transit time is computed as the difference between the abscissas of the two peaks. Otherwise, the transit time is not calculated and the next peak on the first photocell signal is considered. If more than one peak is found in the selection window, the transit time considered is the shortest, as the corresponding peak must be the closest in time to that of the first photocell track.

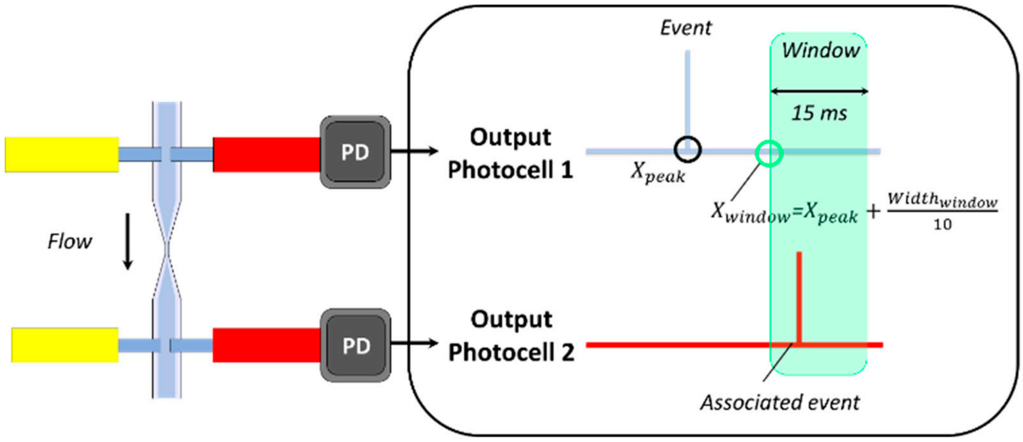

Figure S2. The transit time computed between two consecutive peaks in the output signal of the two photocells. To correctly identify two subsequent events and associate them with the passage of a cell through the bottleneck, an acceptance window must be defined. This eliminates both overlapping peaks – i.e. when only the second photocell detects a flowing cell – and peaks that are not correlated – i.e. when only the first photocell detects cell passage.

2. Temporal Resolution

To fully characterize the temporal resolution of the device, a few aspects must be considered. The operating principle of the chip is based on the measurement of the cell transit time, computationally obtained as the difference in the temporal position of two events (cell passage in the mainstream flow). Each event produces a peak on the corresponding photocell-based detector. The transit time processing algorithm is able to provide valuable data if it can correctly identify the different events (peaks). The width of the resulting single peak, which is called the obscuration time, is a function of the size of the cell while the rise time of the peak is related to the speed of the flowing cell. The minimum obscuration time that can be detected by the device is closely related to the hardware of the signal detection/acquisition system used. To detect it correctly, the bandwidth of the equivalent circuit of the photodetector must first be adequate. It is calculated as

$$f_{BW} = \frac{1}{2\pi C_j R_L}$$

where C_j=1.73pF is the junction capacitance of the diode and R_L is the load resistance. Our values give a bandwidth of approximately 18 MHz. Considering the relationship between the bandwidth and the peak rise(fall) time ($f_{BW}=0.35/t_{rise}$), the photodetector electronic circuit can accurately follow ultra-fast dynamics with rise(fall) times of 20ns. Unfortunately, this cannot yet be the actual resolution, as the sampling rate of the DAQ has to be taken into account. According with our DAQ best performance, we can estimate an obscuration time resolution of about 1 μs (at least 4-5 sampling points for adequate peak identification). Peaks of shorter duration may be lost or not acquired faithfully, affecting the transit time calculation. Therefore, higher resolution can be achieved with the same photodetectors, but with a DAQ that performs better in terms of sample and hold rate parameters.

Transit time resolution is a different matter. Although theoretically the transit time obtained as the time difference between two events may be infinitesimal, experimentally it has proved to be less straightforward. The ability to identify statistically significant differences in median transit time between populations of cells with different elasticities is largely affected by the dispersion (width) of the Gaussian fit that best interpolates the data calculated by the algorithm (Fig. 5). The abscissa position of its maximum represents the median transit time. The high variability of the different transit times within the same population, due both to the heterogeneity of the cell dimension and to the different trajectories followed, takes us away from the theoretical resolution. In our case, a statistically significant value has been between 0.2 and 0.3 ms.
